# DLDTI: a learning-based framework for drug-target interaction identification using neural networks and network representation

**DOI:** 10.1186/s12967-020-02602-7

**Published:** 2020-11-13

**Authors:** Yihan Zhao, Kai Zheng, Baoyi Guan, Mengmeng Guo, Lei Song, Jie Gao, Hua Qu, Yuhui Wang, Dazhuo Shi, Ying Zhang

**Affiliations:** 1 Department of Graduate School, Beijing University of Chinese Medicine, Beijing, China; 2 School of Computer Science and Engineering, Central South University, Changsha, China; 3 National Clinical Research Center for Chinese Medicine Cardiology, Xiyuan Hospital, Cardiovascular Diseases Center, China Academy of Chinese Medical Sciences, Beijing, China; 4 Institute of Cardiovascular Sciences, Health Science Center, Peking University, Key Laboratory of Molecular Cardiovascular Sciences, Ministry of Education, Beijing, China

**Keywords:** Drug-target interaction, Heterogeneous information, Network representation learning, Stacked auto-encoder, Deep convolutional neural networks, Atherosclerosis

## Abstract

**Background:**

Drug repositioning, the strategy of unveiling novel targets of existing drugs could reduce costs and accelerate the pace of drug development. To elucidate the novel molecular mechanism of known drugs, considering the long time and high cost of experimental determination, the efficient and feasible computational methods to predict the potential associations between drugs and targets are of great aid.

**Methods:**

A novel calculation model for drug-target interaction (DTI) prediction based on network representation learning and convolutional neural networks, called DLDTI, was generated. The proposed approach simultaneously fused the topology of complex networks and diverse information from heterogeneous data sources, and coped with the noisy, incomplete, and high-dimensional nature of large-scale biological data by learning the low-dimensional and rich depth features of drugs and proteins. The low-dimensional feature vectors were used to train DLDTI to obtain the optimal mapping space and to infer new DTIs by ranking candidates according to their proximity to the optimal mapping space. More specifically, based on the results from the DLDTI, we experimentally validated the predicted targets of tetramethylpyrazine (TMPZ) on atherosclerosis progression in vivo.

**Results:**

The experimental results showed that the DLDTI model achieved promising performance under fivefold cross-validations with AUC values of 0.9172, which was higher than the methods using different classifiers or different feature combination methods mentioned in this paper. For the validation study of TMPZ on atherosclerosis, a total of 288 targets were identified and 190 of them were involved in platelet activation. The pathway analysis indicated signaling pathways, namely PI3K/Akt, cAMP and calcium pathways might be the potential targets. Effects and molecular mechanism of TMPZ on atherosclerosis were experimentally confirmed in animal models.

**Conclusions:**

DLDTI model can serve as a useful tool to provide promising DTI candidates for experimental validation. Based on the predicted results of DLDTI model, we found TMPZ could attenuate atherosclerosis by inhibiting signal transductions in platelets. The source code and datasets explored in this work are available at https://github.com/CUMTzackGit/DLDTI.

## Background

Research on drug development is becoming increasingly expensive, while the number of newly approved drugs per year remains quite low [1, 2]. In contrast to the classical hypothesis of “one gene, one drug, one disease”, drug repositioning aims to identify new characteristics of existing drugs [3]. Considering the available data on safety of already-licensed drugs, this approach could be advantageous compared with traditional drug discovery, which involves extensive preclinical and clinical studies [4]. Currently, a number of existing drugs have been successfully tuned to the new requirements. Methotrexate, an original cancer therapy, has been used for the treatment of rheumatoid arthritis and psoriasis for decades [5]. Galanthamine, an acetylcholinesterase inhibitor for treating paralysis, has been approved for Alzheimer’s disease [6].

Besides the evidence based on biological experiments and clinical trials, computational methods could facilitate high-throughput identification of novel target proteins of known drugs. To discover targets of drugs with known chemical structures, the prediction of drug-target interaction (DTI) based on numerous computational approaches have provided an alternative to costly and time-consuming experimental approaches [7]. In the past years, DTI prediction has bolstered the identification of putative new targets of existing drugs [8]. For instance, the computational pipeline predicted that telmisartan, an angiotensin II receptor antagonist, had the potential of inhibiting cyclooxygenase. In vitro experimental evidence also validated the predicted targets of this known drug [9]. Further, combined with in silico prediction, in vitro validation and animal phenotype model demonstrated that, topotecan, a topoisomerase inhibitor also had the potential to act as a direct inhibitor of human retinoic-acid-receptor-related orphan receptor-gamma t (ROR-γt) [10].

Most existing prediction methods mainly extract information from complex networks. Bleakley et al. [11] proposed a support vector machine-based method for identifying DTI based on bipartite local model (BLM). Mei et al. [12] proposed BLMNII method for predicting DTIs based on the bipartite local model and neighbor-based interaction-profile inference. In addition, some researchers adopted kernelized Bayesian matrix factorization to predict DTIs, called KBMF2K [13]. A key step of KBMF2K is utilizing dimensional reduction, matrix factorization, and binary classification. Although homogenous network-based derivation methods have achieved good results, they are less effective in low-connectivity (degree) drugs for known target networks. The introduction of heterogeneous information can provide more perspective for predicting the potential of DTI. Recently, Luo et al. proposed a heterogeneous network-based unsupervised method for computing the interaction score between drugs and targets, called DTInet [9]. Subsequently, they proposed a neural network-based method [14] for improving the prediction performance of DTI. Effective integration of large-scale heterogeneous data sources is crucial in academia and industry.

Tetramethylpyrazine (TMPZ) is a member of pyrazines derived from *Rhizoma Chuanxiong* [15]. According to a recent review, TMPZ could attenuate atherosclerosis by suppressing lipid accumulation in macrophages [16], alleviation of lipid metabolism disorder [17], and attenuation of oxidative stress [18]. However, since atherosclerosis is a chronic illness involving multiple cells and cytokines [19], besides lipoprotein metabolism and oxidative stress, other possible targets of TMPZ on atherosclerosis remain unexplored.

In this study, a novel model for prediction of DTI based on network representation learning and convolutional neural networks, referred to as DLDTI is presented for in silico identification of target proteins of known drugs. New DTIs were inferred by integrating drug- and protein-related multiple networks, to demonstrate the DLDTI's ability of integrating heterogeneous information and neural networks to extract deep features of drugs and target networks as well as attributes to effectively improve prediction accuracy. Moreover, comprehensive testing demonstrated that DLDTI could achieve substantial improvements in performance over other prediction methods. Based on the results predicted by DTDTI, new interactions between TMPZ and targets involved in atherosclerosis, namely signal transduction in platelets, were validated in vivo. The anti-atherosclerosis effect of TMPZ was confirmed in a novel atherosclerosis model. In summary, these improvements could advance studies on drug-target interaction.

## Methods

### Prediction experiments

#### Human drug-target interactions database

In this study, we use the DrugBank established by Wishart et al*.* as the benchmark dataset, which can be downloaded at https://www.drugbank.ca [20]. The chemical structure of each drug in SMILES format is extracted from and extracted from DrugBank. In the experiments, only those that satisfied the human target represented by a unique EnsemblProt login number were used. In detail, 904 drugs and 613 unique human targets (proteins) were linked to construct a DTI network $A$ as positive samples, and a matching number of unknown drug-target pairs (by excluding all known DTIs) were randomly selected as negative samples. The labels of training set and testing set are binary label.

#### Feature representation

##### Gaussian interaction profile kernel similarity for drugs and targets

On the basis of previous work, drug similarity can be measured by calculating nuclear similarity through Gaussian interaction profile (GIP) kernel similarity [21, 22]. The GIP similarity between drug ${d}_{i}$ and drug ${d}_{j}$ is defined as follow:

1 $$ {\text{D}}_{sim} \left( {d_{i} ,d_{j} } \right) = exp\left( { - \tau_{d} *\left| {\left| {V\left( {d_{i} } \right) - V\left( {d_{j} } \right)} \right|} \right|^{2} } \right) $$

where the binary vector $V\left({d}_{i}\right)$ and $V\left({d}_{j}\right)$ is the *i*-th row vector and the *j*-th row vector of the drug-target interaction network $A$. The parameter ${\tau }_{d}$ is the kernel bandwidth. It computes by normalizing original parameter $\tau_{d} ^{\prime}$:

2 $$ \tau_{d} = \frac{{\tau^{\prime}_{d} }}{{\frac{1}{{n_{d} }}\mathop \sum \nolimits_{i = 1}^{{n_{d} }} \left| {\left| {V\left( {d_{i} } \right)} \right|} \right|^{2} }} $$

Similarly, the GIP similarity for targets can be defined as follows:

3 $$ {\text{D}}_{sim} \left( {d_{i} ,d_{j} } \right) = exp\left( { - \tau_{d} *\left| {\left| {V\left( {p_{i} } \right) - V\left( {p_{j} } \right)} \right|} \right|^{2} } \right) $$

where the binary vector $V\left({p}_{i}\right)$ and $V\left({p}_{j}\right)$ is the *i*-th row vector and the *j*-th column vector of the drug-target interaction network $A$. The parameter ${\tau }_{p}$ is the kernel bandwidth. It computes by normalizing original parameter $\tau_{p} ^{\prime}$:

4 $$ \tau_{p} = \frac{{\tau^{\prime}_{p} }}{{\frac{1}{{n_{p} }}\mathop \sum \nolimits_{i = 1}^{{n_{p} }} \left| {\left| {V\left( {p_{i} } \right)} \right|} \right|^{2} }} $$

##### Protein sequence feature

The sequences for drug targets (proteins) in *Homo sapiens* downloaded from the String database ( https://string-db.org/) [24]. The *k-mer* algorithm is used to count Subsequence information in protein sequences and uses it as a feature vector to solve the alignment problem posed by differences in sequence length [24].

##### Drug structure feature

The SMILES for drugs downloaded from the DrugBank database. We use Morgan fingerprint, a circular fingerprint, to map the structure information of drugs to feature vectors.

##### Graph embedding-based feature for drugs and targets

Graph data is rich in behavioral information about nodes, and behavioral information can be used as a descriptor to describe drugs and targets that can be more comprehensive description of the characteristics [25]. So how do we map a high-dimensional dense matrix like graph data to a low-density vector? Here we introduce the Graph Factorization algorithm [26]. Graph factorization (GF) is a method for graph embedding with time complexity O(|E|). To obtain the embedding, GF factorizes the adjacency matrix of the graph to minimize the loss functions as follow:

5 $$ \varepsilon \left( {P,Q,\lambda } \right) = \frac{1}{2}\mathop \sum \limits_{{\left( {i,j} \right) \in E}} \left( {P_{ij} - \left\langle {Q_{i} ,Q_{j} } \right\rangle } \right)^{2} + \frac{\lambda }{2}\mathop \sum \limits_{i} \left| {\left| {Q_{i} } \right|} \right|^{2} $$

where $\lambda$ is the regularization coefficient. $P$ and $Q$ are the adjacency matrix with weights and factor matrix, respectively. $E$ is the set of edges, which includes *i* and *j*.

The gradient of the function $\varepsilon$ with respect to ${Q}_{i}$ is defined as follow:

6 $$ \frac{\partial \varepsilon }{{\partial Q_{i} }} = - \mathop \sum \limits_{{k \in N_{o} }} \left( {P_{ij} - \left\langle {Q_{i} ,Q_{j} } \right\rangle } \right)Q_{j} + \lambda Q_{i} $$

where ${N}_{o}$ is the set of neighbors of node $o$. With the Graph Factorization algorithm, graph embeddings of drugs and targets in the drug-target interaction network can be obtained to describe their behavioral information.

#### Stacked autoencoder

As DLDTI integrates heterogeneous data from multiple sources, including protein sequence information, drug structure information, and drug-target interaction network information, the integrated biological data suffers from noise, incomplete and high-dimensional. Here, the stack autoencoder (SAE) is introduced to find the optimal mapping of drug space to target space to obtain low dimensional drug Feature vector [27, 28]. SAE can be defined as follows:

7 $$ y = f\left( x \right) = S_{e} \left( {W + b} \right) $$

8 $$ z = g\left( y \right) = S_{d} \left( {W^{\prime}y + b^{\prime}} \right) $$

where $y$ and $z$ are encoding function and decoding function respectively. $W$ and ${W}^{^{\prime}}$ are the relational parameters between two layers. $b$ and ${b}^{^{\prime}}$ are vectors of bias parameters. The activation function used is ReLU:

9 $$ S_{e} \left( t \right) = S_{d} \left( t \right) = max\left( {0,W^{T} + b} \right) $$

#### Convolutional neural network

Lecun et al. proposed convolutional neural networks in 1989 [29]. Subsequently, they have performed well in tasks such as image classification, sentence classification, and biological data analysis. Thus, in this study, convolutional neural networks were used to train supervised learning models to predict potential DTIs. In this work, convolutional neural networks were chosen as supervised learning models to learn deep features and predict potential DTIs. The model used includes convolutional and activation layers, a Maxpooling layer, a fully connected layer and a softmax layer. Their roles are, respectively, to extract depth features, down-sample, and classify samples. The convolutional layer is one of the most important parts of the CNN and aims to learn the deep characteristics of the input vectors, which is defined as follows

10 $$ C_{m} = \mathop \sum \limits_{i = 1}^{{N_{k} }} W_{i} X_{m + j} $$

where $X$ is the input feature of length$L$. ${N}_{k}$ is the number of kernels.$m\in \{0, ... , L-N\}$, W is a weight vector of length${N}_{k}$. Then, the feature map ${C}_{m}$ is put into the activation function ReLU, which is defined as follow:

11 $$ f\left( x \right) = max\left( {0,x} \right) $$

The role of the ReLU function is to increase the nonlinear relationship between the layers of the neural network, save computation, solve the gradient disappearance problem, and reduce the interdependence of parameters to mitigate the overfitting problem.

The convolutional and maximum pooling layers can extract important features from the input vectors. The output of all kernels is then concatenated into a vector and fed to the fully-connected layer $f\left(W\cdot y\right)$. Where $y$ is the output of Maxpooling layer and $W$ is the weight matrix. Finally, the softmax layer scores the input vectors as a percentage.

#### Pathway analysis of predicted results from DLDTI

Atherosclerosis-related gene sets were collected from GeneCards (https://www.genecards.org/) [30]. After using retrieve tool on Uniprot database (https://www.uniprot.org/), different identifiers from Drug Bank and GeneCards were converted to UniProtKB. Based the intersection of potential targets of TMPZ from DLDTI model and confirmed target proteins of atherosclerosis, the matched targets were regarded as the predicted targets of TMPZ on atherosclerosis. The predicted targets were uploaded to the Search Tool for the Retrieval of Interacting Genes/Proteins database (STRING, Version 11) (https://string-db.org/) [23] for Kyoto Encyclopedia of Genes and Genomes (KEGG) pathway and Gene Ontology (GO) biological process analysis.

### Validation experiments

#### Ldlr−/− hamsters

This study was approved by the Animal Ethics Committee of Xiyuan Hospital and strictly adhered to the principles of laboratory animal care (NIH publication No.85Y23, revised 1996). Male, 8 week aged and low-density lipoprotein receptor knock-out (Ldlr−/−) hamsters were provided by the health science center, Peking University. The Ldlr−/− genotype was confirmed using polymerase chain reaction (PCR) analysis of DNA extracts from ears [31]. After 1 week of acclimatization, they were fed on high-cholesterol and high-fat (HCHF) diet containing 15% lard and 0.5% cholesterol (Biotech company, China) for 8 weeks. The Ldlr−/− hamsters were then randomly divided into three groups according to their weights (n = 8 per group) and orally administered with a mixture of volume vehicle (distilled water), TMPZ (32 mg/kg/d) and clopidogrel (32 mg/kg/d) drugs for 8 weeks. Wild type (WT) golden Syrian hamsters (n = 8) purchased from Vital River Laboratory (Charles River, Beijing, China) were fed on a standard chow diet as healthy control. All hamsters were maintained on a 12 h light/12 h dark cycle with free access to water.

Hamsters were fasted for 12 h and anesthetized by intraperitoneal injection of 1% sodium phenobarbital (70 mg/kg). Blood samples were taken from abdominal aortas and plasma was separated by centrifugation for 10 min at 2700 × *g*. TC, TG and HDL were determined using commercially available kits (BIOSINO, China).

#### Oil red O staining

As described previously [32], anesthetized hamsters were perfused with 0.01 M PBS through the left ventricle. In brief, hearts and whole aortas were placed in 4% paraformaldehyde solution overnight, transferred to 20% sucrose solution for 1 week. Hearts were then fixed into O.C.T compound and cross-sectioned (8 μm per slice). The atherosclerotic lesions in aortic root were stained with 0.3% Oil red O solution (Solarbio, China), rinsed with 60% isopropanol and distilled water and counterstained with hematoxylin. The results were represented by the percentage positive area of total area (*en face* analysis) and net lesion area (aortic root sections). Images were analyzed with Image J [33].

#### Histological analysis

Analysis of atherosclerotic plaque cell composition was determined by immunohistochemistry (IHC) analysis of the aortic root. Macrophages and smooth muscle cells (SMC) were stained with CD68 (BOSTER, BA36381:100) antibody and a-SMA antibody (BOSTER, A03744, 1:100) as reported previously in hamster researches [31]. Then biotinylated second antibody (Vector Laboratories, ABC Vectastain, 1:200) were used for incubation under 2% normal blocking serum. The cryosections were visualized using 3,3-diaminobenzidine (Vector Laboratories, DAB Vectastain). The results were represented by the percentage positive area of total cross-sectional vessel wall area in the aortic root sections and analyzed using Image J [33].

#### Washed platelet preparation

Blood per hamster, 3 to 4 mL was collected from abdominal aortas into a tube containing an acid-citrate-dextrose anticoagulant (83.2 mM D-glucose, 85 mM trisodium citrate dihydrate, 19 mM citric acid monohydrate, pH5.5). Platelet-rich plasma (PRP) was prepared after centrifugation at 300 × *g* for 10 min in room temperature. For washed platelet preparation, PRP was centrifuged at 1500 × *g* for 2 min. After collecting supernatant consisting of platelet-poor plasma into another centrifuge tube, the remaining PRP was washing three times, and the pellet was re-suspended in a modified Tyrode buffer (2.4 mM HEPES, 6.1 mM D-glucose, 137 mM NaCl, 12 mM HaHCO3, 2.6 mM KCl, pH7.4).

#### Assessment of platelet activity

Washed platelets were loaded with fura-2/AM(5 μM, Molecular Probe) in the presence of Pluronic F-127 (0.2 μg/mL, Molecular Probe) and then incubated at 37 °C for 1 h in the dark [34]. Platelets were washed and re-suspended in Tyrode buffer containing 1 mM calcium. After activation of ADP (20 μM, Sigma), intracellular calcium concentration was measured using a fluorescence mode of Synergy H1 microplate reader (Biotek, USA). Excitation wavelengths was alternated at 340 and 380 nm. Excitation was measured at 510 nm. TritonX-100 and EGTA were used for calibration of maximal and minimal calcium concentrations, respectively. Washed platelets were activated by ADP and then lysed by 0.1 M HCl on ice. According to the manufacturer’s instructions, the level of intracellular cAMP was determined by ELISA (Enzo Life Sciences, ADI-900-066).

### Western blot analysis

Washed platelets from each group were lysed with radioimmunoprecipitation assay buffer with the presence of protease and phosphatase inhibitor mixtures on ice (Solarbio, China). Lysates were separated by 10,000 × *g* centrifugation for 10 min at 4 °C. Total protein concentrations were determined by BCA method. Equal amounts of total protein (40 μg) were resolved in SDS-PAGE and electroblotted. The nitrocellulose membranes were blocked with 5% skimmed milk at room temperature for 2 h and incubated with primary antibodies targeting PI3K(CST, 4257 T, 1:500), Akt(CST, 9272, 1:2000), p-Akt(CST,2965,1:1000) and GADPH (Abcam, ab8245, 1:5000) overnight at 4 °C. The membranes were then incubated with the HRP-conjugated anti-rabbit antibody for 1 h at 37 °C, followed by enhanced chemiluminescence detection.

### Statistical analysis

All data were expressed as mean ± standard error. Shapiro-Wild test and Levene’s test were used for normality of data distribution and homogeneity of variances, respectively. An unpaired student’s *t*-test were used to compare data in different groups when data normally distributed and variances were equal among groups. Unpaired t test with Welch’s correction were used when unequal standard deviation among groups. Mann–Whitney test were used for nonparametric test. All *p* values less than 0.05 were considered statistically significant. All statistical analyses were performed using GraphPad Prism 8.0 (GraphPad, United states).

## Results

### Overview of DLDTI and performance evaluation on predicting drug-target interaction

**Fig. 1:**
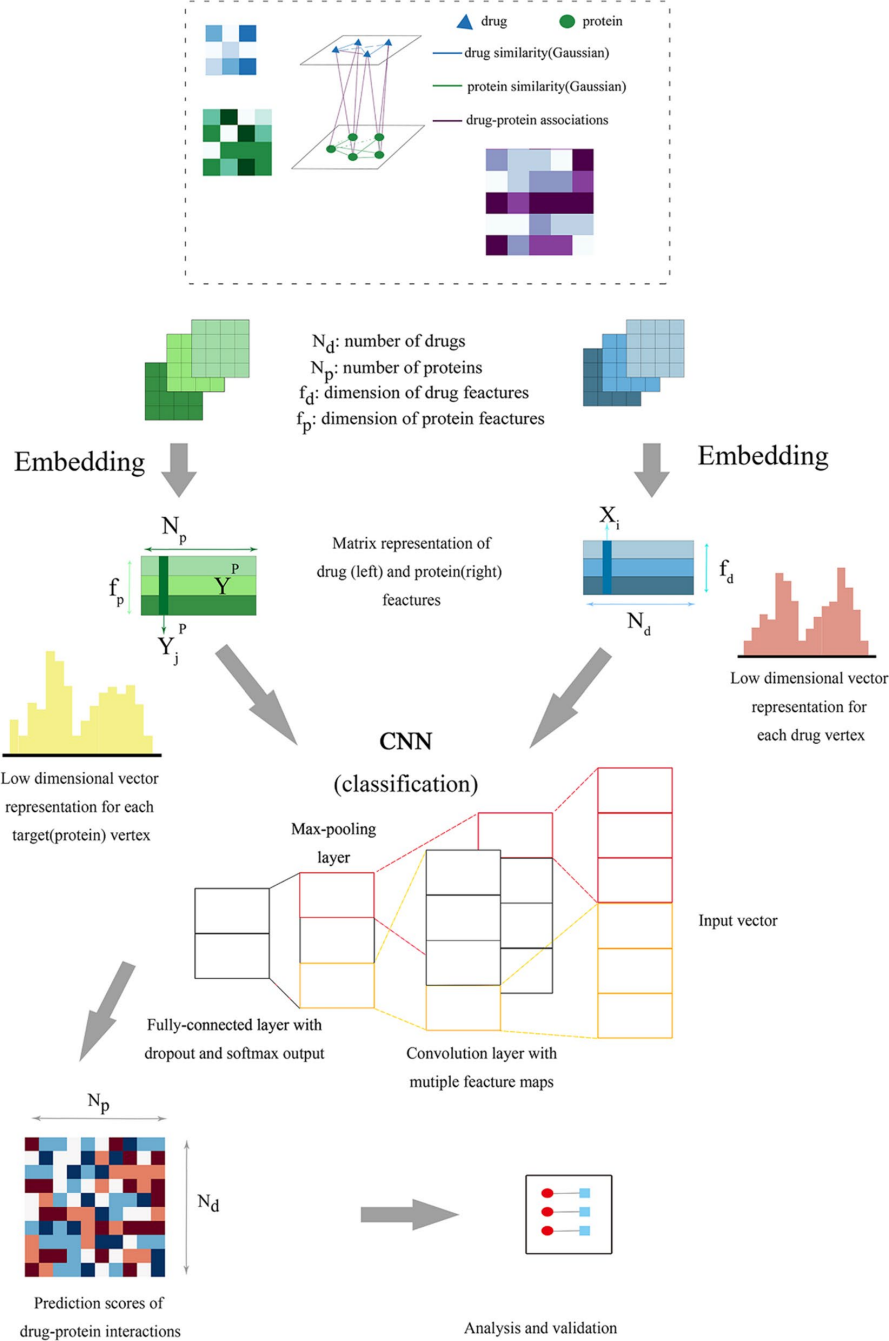
The flowchart of the DLDTI pipeline. DLDTI first integrates a variety of drug-related information sources to construct a heterogeneous network and applies a compact feature learning algorithm to obtain a low-dimensional vector representation of the features describing the topological properties for each node. Next, DLDTI determines the optimal mapping from the plenary mapping space to the prediction subspace, and whether the feature vector is close to the known correlations. Afterwards, DLDTI infers the new DTIs by ranking the candidates according to their proximity to the predicted subspace

**Fig. 2:**
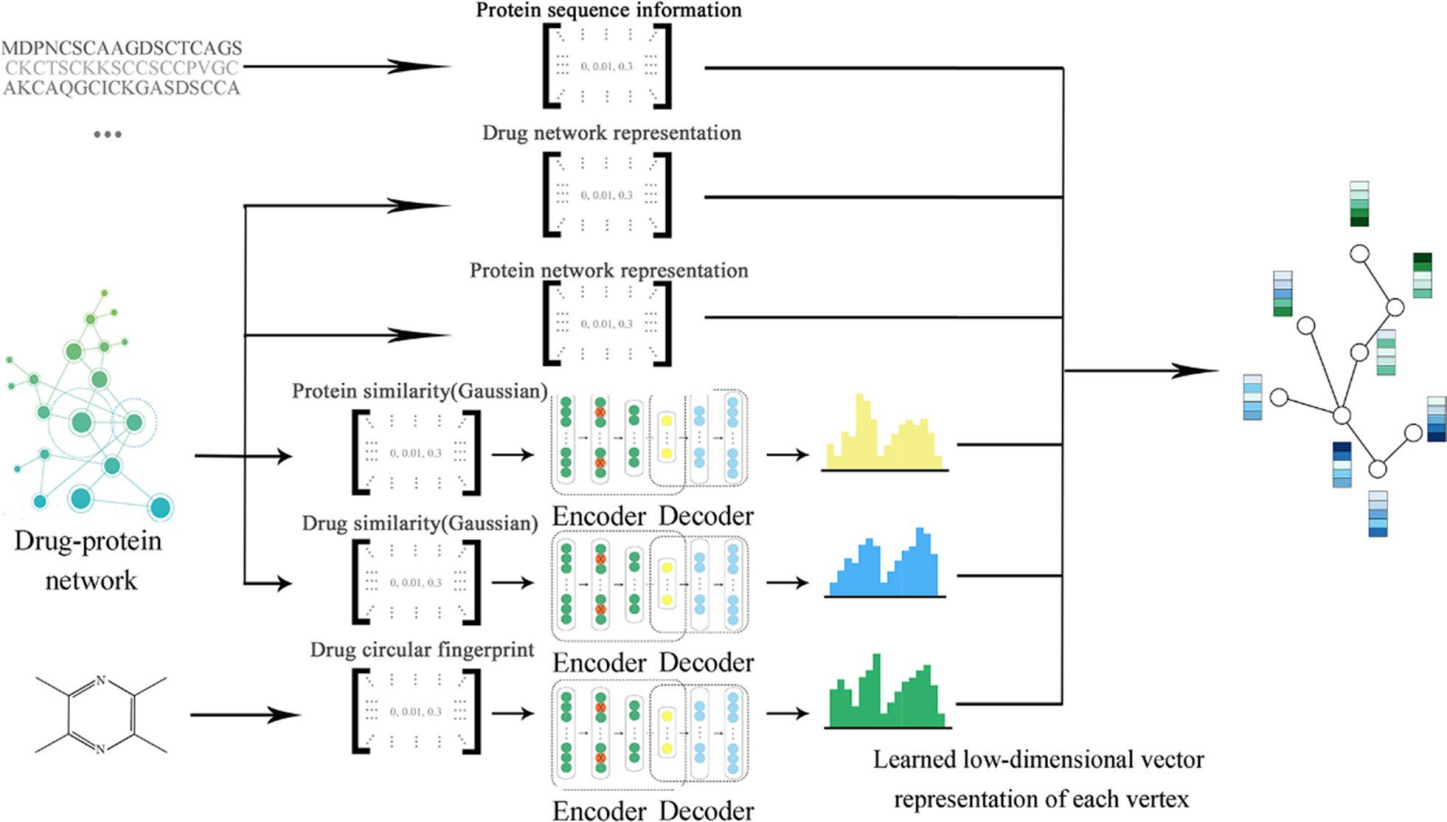
Schematic illustration of compact feature learning. The Node2Vec algorithm is firstly used to calculate the topology information in complex networks. GIP kernel similarity and drug structure information are then extracted by a stacked automatic encoder, and the heterogeneous information is integrated to obtain a low-dimensional representation of the feature vector of each node. The resulting low-dimensional descriptor integrates the attribute characteristics, interaction information, relationship attributes and network topology of each protein or target node in the complex network

A new computational model referred to as DLDTI was developed to predict potential DTIs to identify novel behavior of traditional drugs based on complex networks and heterogeneous information. As an overview (Fig. 1), DLDTI integrates learning from complex network's various heterogeneous information to obtain low-dimensional and deep rich features (Fig. 2), through a processing method known as compact feature learning. During compact feature learning, the resulting low-dimensional descriptor integrates attribute characteristics, interaction information, relational properties, and network topology of each protein or target node in the complex network. DLDTI then determines the optimal mapping from the plenary mapping space to the prediction subspace, and whether the feature vector is close to the known correlations. Afterwards, DLDTI infers the new DTIs by ranking the DTI candidates according to their proximity to the predicted subspace.

**Fig. 3:**
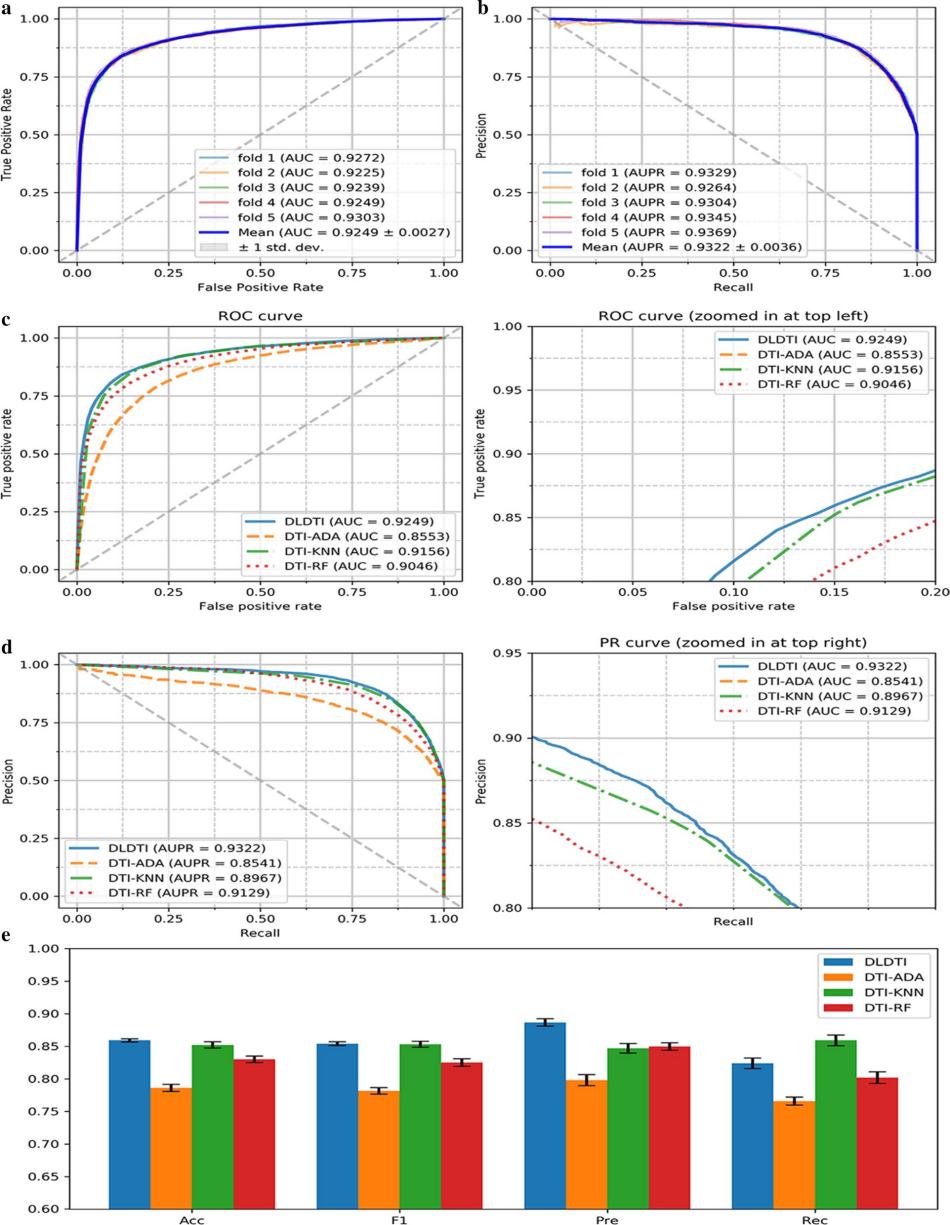
Performance of DLDTI. **a** ROC curves performed by DLDTI model on DrugBank dataset. **b** PR curves performed by DLDTI model on DrugBank dataset. **c** Performance comparison (AUC scores) among four different prediction model which are DTI-ADA, DTI-KNN, and DTI-RF. **d** Performance comparison (AUPR scores) among four different prediction models including DTI-ADA, DTI-KNN, and DTI-RF. **e** Performance comparison (Acc., F1, Pre., Rec. scores) among DTI-ADA, DTI-KNN, and DTI-RF prediction models

DLDTI yields accurate DTI prediction. Firstly, the predictive performance of DLDTI was assessed using five-fold cross-validation, where randomly selected subset of one-fifth of the validated DTI were paired with an equal number of randomly sampled non-interacting pairs to derive the test set. The remaining 75% of known DTI and same number of randomly sampled non-interacting pairs were used to train the model. DLDTI was compared with three methods based on different classifiers used for DTI prediction, including DTI-ADA, DTI-KNN, and DTI-RF [35–37]. The comparison revealed that DLDTI consistently outperforms the other three methods, with 0.93% higher AUC, 3.55% higher AUPR, 0.61% higher accuracy (Acc), 3.96% higher precision (Pre) than the second-best method (Fig. 3c–e). Compared to DTI-ADA (which predicts DTI based on the AdaBoost classifier), the DLDTI of the area under AUROC and AUPR was 6.96 and 7.81% higher, respectively, which could have been due to the inability of traditional machine learning to extract deeper abstract features for prediction, resulting in poor performance, while DLDTI applies a deep convolutional neural network approach and is able to capture the potential structural properties of complex networks and heterogeneous information.

### Enrichment analysis suggested TMPZ might affect signal transduction pathways involved in platelet activation

To elucidate the potential function of TMPZ on atherosclerosis, the predicted results from DLDTI model were uploaded to the search tool for retrieval of interacting genes/proteins database (STRING) to determine over-represented KEGG pathways and GO categories. GO analysis demonstrated that 31.4% of genes were involved in signal transduction (Additional file 1: Table S1). As shown in Table 1, PI3K/Akt signaling pathway, neuroactive ligand-receptor interaction, MAPK signaling pathway, calcium signaling pathway, Rap1 signaling pathway, cGMP-PKG signaling pathway, and cAMP signaling pathway were the top-ranked results of KEGG enrichment. It is noteworthy that ADP-mediated platelet activation via purinergic receptors included almost all signal transduction pathways shown in Table 1 [38, 39]. Interestingly, among the 288 predicted targets of TMPZ on atherosclerosis, 190 proteins were also involved in the platelet activation process (Additional file 2: Table S2). Therefore, it was assumed that the anti-atherosclerosis potential of TMPZ could be largely attributed to its inhibition of purinergic receptor-dependent platelet activation, which involves signal transduction pathways such as PI3K/Akt. Based on the predicted result, clopidogrel, an anti-platelet drug widely used in the clinical application, was chosen as the positive control.

**Table 1:** KEGG pathway enrichment analysis of DLDTI results

Class	KEGG term	Count	*P* value
Signal transduction	PI3K-Akt signaling pathway	36	2.49E−17
	Neuroactive ligand-receptor interaction	32	6.04E−17
	MAPK signaling pathway	29	1.08E−13
	Calcium signaling pathway	26	1.01E−15
	Rap1 signaling pathway	22	2.99E−11
	cGMP-PKG signaling pathway	20	2.99E−11
	cAMP signaling pathway	16	3.83E−07
Metabolism	Metabolism of xenobiotics by cytochrome P450	23	4.27E−20
	Steroid hormone biosynthesis	17	1.28E−14
	Retinol metabolism	15	5.89E−12
Immune system	Complement and coagulation cascades	21	3.06E−17
	Th17 cell differentiation	15	1.77E−09
Others	Regulation of actin cytoskeleton	16	6.90E-07
	Gap junction	15	2.74E-10
	Fluid shear stress and atherosclerosis	15	2.91E-08

### Validation

#### Ldlr−/− hamsters developed severe hyperlipidemia and atherosclerosis lesions when fed with HFHC diet

**Fig. 4:**
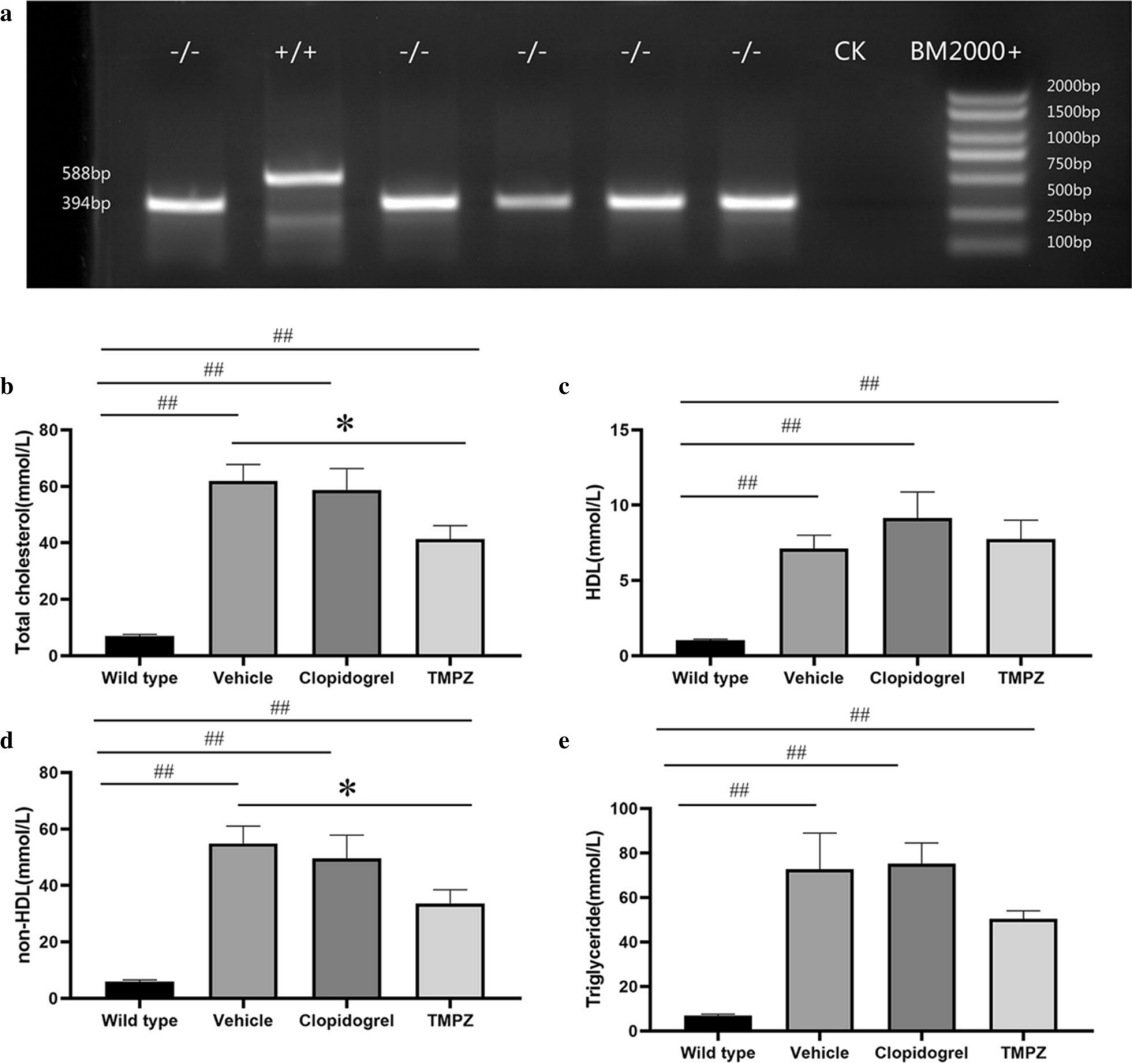
Genotyping and lipid parameters between different groups. **a** PCR analysis was performed using ear genomic DNA from WT (+ / +) and homozygote (− / −) with the Δ194 deletion. The concentrations of plasma TC **b**, HDL (**c**), non-HDL (**d**) and TG (**e**) were measured in WT, vehicle, TMPZ and clodipogrel groups at the endpoint of this experiment. Differences were assessed by unpaired student’s *t*-test or Mann–Whitney test. *p < 0.05 vs Vehicle, **p < 0.01 vs Vehicle. ^##^*p* < 0.01 vs WT

Before dietary induction, genotypes were determined by PCR analysis. Using ear genomic DNA, 194-nucleotide deletion (Δ194) was detected in homozygous (−/−) hamsters **(**Fig. 4a). After feeding them on HCHF diet for 16 weeks, Ldlr−/− hamsters developed severe hyperlipidemia. As an antiplatelet medication, clopidogrel did not influence circulating levels of TC, TG, HDL and non-HDL (Fig. 4b–e). Compared with vehicle-treated hamsters, decreased levels of TC (*p* < 0.05) and non-HDL (*p* < 0.05) were observed in TMPZ-treated group (Fig. 4b and d). However, TMPZ did not influence TG or HDL levels.

#### TMPZ ameliorated atherosclerosis lesion progression

**Fig. 5:**
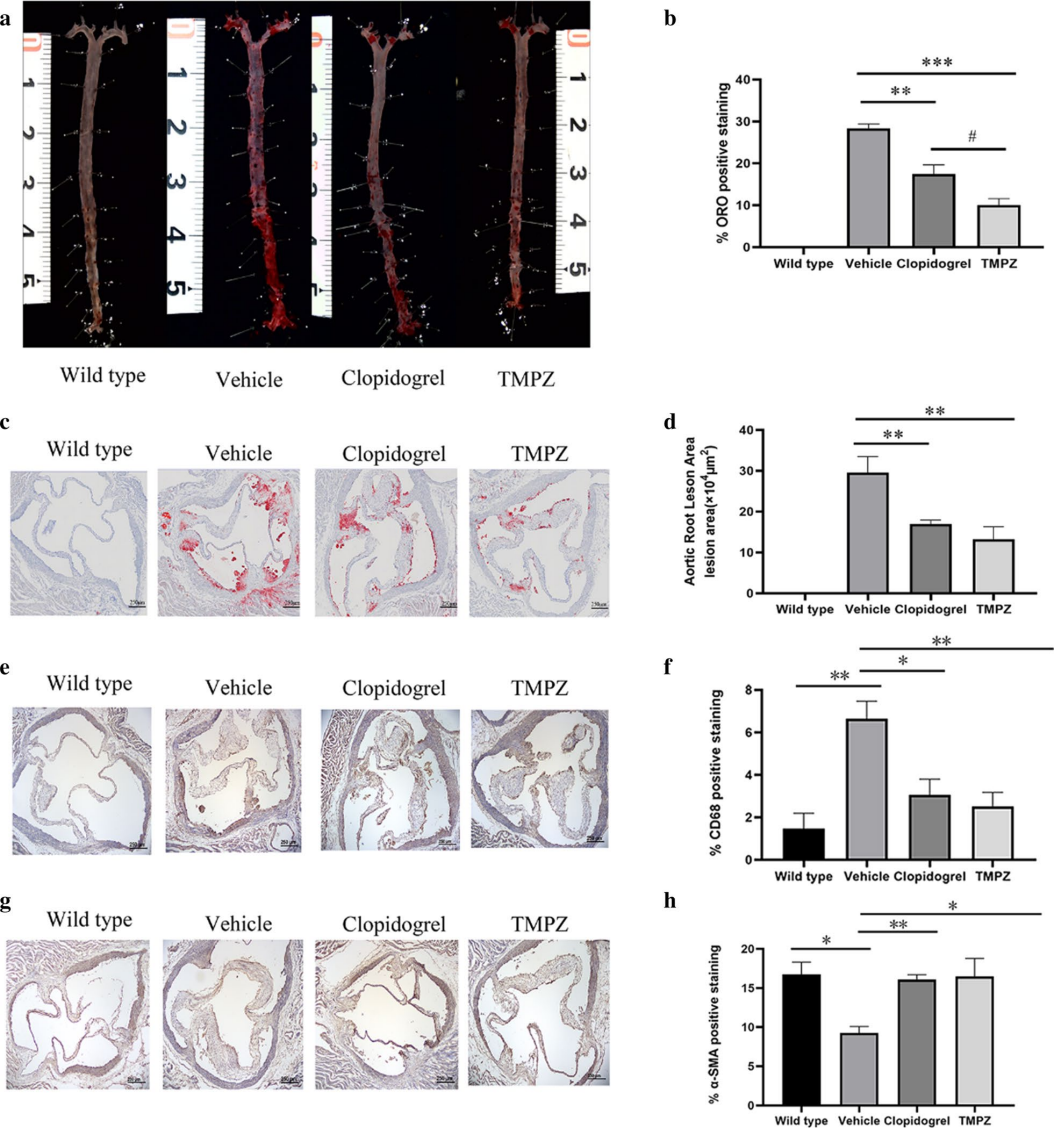
Histological analysis. **a** Representative images of *en face* analysis. n = 6. **b** Quantitative analysis of lesion areas in whole aortas. Differences were assessed by unpaired students’ *t*-test. **c** Representative images of Oil Red O staining of aortic root sections. **d** Quantitative analysis of lesion areas in aortic root sections. **e** Representative images of macrophage (CD68) analysis (**b**) Quantitative analysis of lesions area in macrophage analysis. **f** Representative images of SMC (SMA) analysis (**g**) Quantitative analysis of lesions area in SMC. Differences were assessed by unpaired students’ *t*-test. *p < 0.05 vs Vehicle, **p < 0.01 vs Vehicle. ^#^*p* < 0.05 vs clopidogrel. Scale bar = 250 μm. n = 3.

The *en face* analysis demonstrated that vehicle-treated hamsters developed significant atherosclerotic lesions (mean value 28.38%) throughout the whole aorta. However, atherosclerotic lesions induced by the same dietary manipulation in TMPZ- and clopidogrel-treated groups were significantly decreased (mean value 10.02% and mean value 17.47%, respectively) (Fig. 5a, b). It’s noteworthy that the lesion area in TMPZ-treated group was also less than that in clopidogrel-treated group (Fig. 5b). As the blank control group, WT hamsters on chow diet did not develop any lesions throughout the aorta.

Similar to the *en face* analysis, the HFHC fed vehicle group had significantly increased lesion areas (mean area 29.58 × 10^4^ μm^2^) in aortic roots compared to the blank controls measured by image analysis of Oil Red O staining, and either TMPZ (mean area 13.25 × 10^4^ μm^2^) or clopidogrel (mean area 16.99 × 10^4^ μm^2^) treatment reduced the lipid-rich areas (Fig. 5c, d).

Under the stimulation of adhesion molecules, monocytes infiltrate into the intima and differentiate into macrophages [40]. Besides macrophage accumulation, diminished SMC could also exacerbate the formation of unstable plaques [41]. To determine the components of atherosclerosis lesions in the aortic root, IHC staining for macrophages and SMC was performed. As shown in Fig. 5e, f, the percentage of macrophage positive staining in lesions was increased by atherosclerosis progression in the vehicle-treated group. WT group (mean value 1.48%) had significantly fewer macrophage accumulation than vehicle-treated group (mean value 6.65%). Infiltrated macrophages in lesions were significantly decreased by TMPZ (mean value 2.52%) or clopidogrel (mean value 3.07%) treatment. As shown in Fig. 5 g,  h, the percentage of a-SMA positive staining was diminished in Ldlr−/− hamsters (mean value 9.27%) compared with the WT hamsters (mean value 16.76%). Administration TMPZ (mean value 16.50%) or clopidogrel (mean value 16.09%) for 8 weeks could ameliorate SMC reduction in atherosclerosis lesions.

#### TMPZ inhibited signaling transduction in ADP-mediated platelet activation

In addition to the surrogates of platelet activation, calcium and cAMP signaling are also essential in signal transduction. Downstream from Gq signaling, protein kinase C activation results in the formation of inositol triphosphate, which leads to an elevation of intracellular calcium [38]. Calcium mobilization is also required for the phosphorylation of Akt (also known as protein kinase B) in PI3K/Akt signaling pathway [42]. In response to ADP, Gi signaling activation mediates the inhibition of AC, resulting in the diminished synthesis of cAMP. The inhibitory effect of Gi on cAMP synthesis could cause platelet activation [39].

**Fig. 6:**
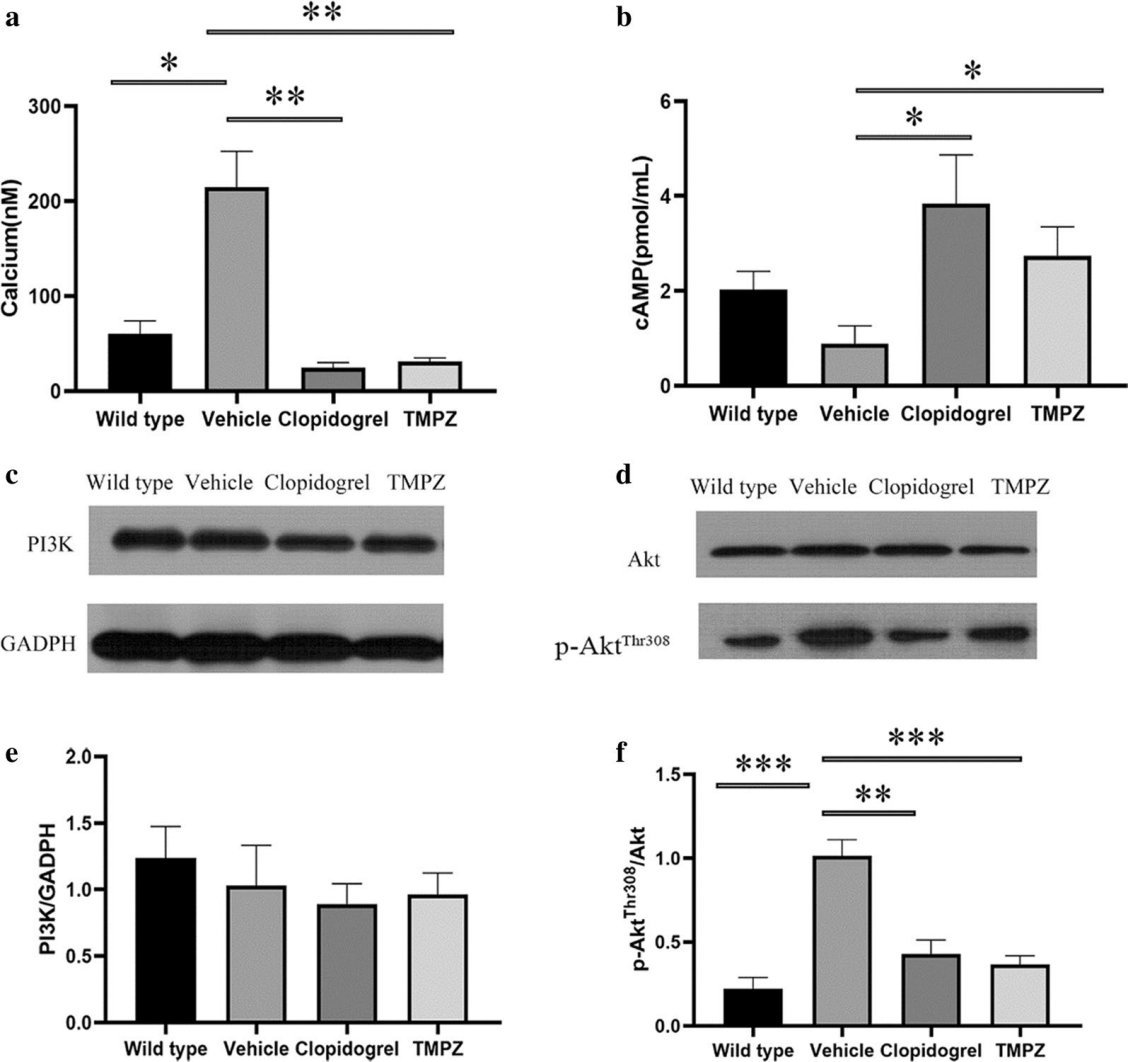
Signaling transduction in ADP-mediated platelet activation. **a** Intracellular calcium concentration. **b** Intracellular cAMP concentration. Western blot analyses of the expression of PI3K (**c**), Akt (**d**) and p-Akt (**d**). Differences were assessed by unpaired student’s *t*-test with or without Welch’s corrections. **p < 0.01 vs Vehicle, *p < 0.05 vs Vehicle. n = 4–6

Figure 6 shows that fura-2/AM is a membrane-permeant calcium indicator. The ratio of F340/F380 is directly correlated to the amount of intracellular calcium. The data revealed that TMPZ and clopidogrel markedly inhibited calcium mobilization, as detected using fluorescence mode of Synergy H1 microplate reader. Moreover, TMPZ-and clopidogrel-treated groups showed a higher concentration of cAMP in the active platelets. These findings indicate that TMPZ and clopidogrel could inhibit calcium mobilization and elevate intracellular concentration of cAMP, thereby inhibiting platelet activation.

As the major downstream effector of PI3K, Akt plays an essential role in the regulation of platelet activation. Stimulation of platelets with ADP could result in Akt activation, which was indicated by Akt phosphorylation [42]. The protein expressions of PI3K, Akt, and p-Akt in the top-ranked signal transduction pathway were measured to validate the predicted pathways. ADP-induced P2Y12 receptor activation could cause PI3K dependent Akt phosphorylation, a critical positive regulator pathway for signal amplification. There was no difference in PI3K expression levels between WT, vehicle, TMPZ, and clopidogrel groups (Fig. 6c). Phosphorylation of Akt was inhibited by TMPZ or clopidogrel administration when compared with vehicle-treated group. It is noteworthy that phosphorylation of Akt did not differ between WT, TMPZ and clopidogrel groups, which indicates that platelet activity in atherosclerosis hamsters treated with TMPZ or clopidogrel could be comparable to that in healthy ones (Fig. 6d). These findings indicate that TMPZ and clopidogrel could attenuate Akt signaling, thereby blocking the platelet activation induced by ADP.

## Discussion

In summary, we provide a novel DTI model and validate its efficacy in animal model. This DLDTI model could provide an alternate to the high-throughput screening of drug targets. The proposed approach simultaneously fuses the topology of complex networks and diverse information from heterogeneous data sources, and copes with the noisy, incomplete, and high-dimensional nature of large-scale biological data by learning the low-dimensional and rich depth features of drugs and proteins. The low-dimensional descriptors learned by DLDTI that capture attribute characteristics, interaction information, relational properties, and network topology attributes for each drug or target node in a complex network. The low-dimensional feature vectors were used to train DLDTI to obtain the optimal mapping space and to infer new DTIs by ranking potential DTIs according to their proximity to the optimal mapping space. We inferred new DTIs by integrating drug- and protein-related multiple networks, demonstrating the DLDTI's ability to integrate heterogeneous information and that deep neural networks are capable of extracting drug and target networks and the deep features of attributes can effectively improve the prediction accuracy. Compared with three methods based on different classifiers used for DTI prediction, including DTI-ADA, DTI-KNN, and DTI-RF [35–37], DLDTI consistently outperforms the other three methods. More importantly, compared to DTI-ADA, the AUROC and AUPR of DLDTI was 6.96% and 7.81% higher. This result could be attributed to the inability of traditional machine learning to extract deeper abstract features for prediction, resulting in poor performance, while DLDTI applies a deep convolutional neural network approach and is able to capture the potential structural properties of complex networks and heterogeneous information.

Furthermore, in the validation study of the DLDTI model, we used TMPZ (a drug with known structure) to explore its effects on atherosclerosis in vivo. Consistent with previous studies [16–18], the results revealed that TMPZ could ameliorate the phenotyping of atherosclerosis in Ldlr−/− hamsters, a novel atherosclerosis model [31, 43]. Diminished lipid deposition and macrophage accumulation, and increased percentage of SMC were observed in TMPZ- and clopidogrel-treated hamsters. Interestingly, the majority of potential pathways of TMPZ on atherosclerosis were involved in signal transduction of platelet activation. From the initial endothelial dysfunction in the early stage to the destabilized plaques in the advanced stage, platelet plays a pivotal role [44]. Activated platelets act as the key trigger for rupture-prone plaque formation. Current evidence shows that platelet hyperactivity is associated with a prothrombotic state and increased incidence of recurrent cardiovascular events among patients with coronary artery disease [45]. Platelets can be activated by various stimuli like collagen, thrombin, and ADP. Based on the pathway analysis of predicted results, this work focused on signal transduction in ADP-mediated platelet activation (Table 1). The results revealed that the activated signal transductions, characterized by increased calcium mobilization, decreased cAMP concentration and increased phosphorylation of Akt were observed in ex vivo platelets from vehicle-treated hamsters, while platelets from TMPZ- and clopidogrel-treated hamsters showed inhibited platelet activation.

A future direction of our study is to solve the “cold-start” problem, which is a challenge that all algorithms that apply collaborative filtering technology will face. In this paper, the feature vectors with the highest ranked protein or drug are weighted, based on the similarity of protein sequences and the similarity of drug structures, to obtain new interaction feature vectors to solve the cold start problem. After experiments, we found that the model works best when the feature vector of the highest ranked protein or drug is weighted by 60, 30, and 10%. Without adverse event databases inserted, although our prediction model is particularly helpful for understanding the unknown pharmacological effects of drugs with known chemical structures, it could offer little help to tell reported DTIs would be beneficial or harmful. As reviewed previously, drug adverse effects are complicated phenomena. It might be difficult to predict adverse effects, only relying on the information of DTIs [46]. The more promising way is to use pharmacological information such as drug side effects and adverse drug reactions. We will consider using multi-task model algorithms and adverse event databases to solve this problem in future work.

In addition, in the validation study, we only examined the top-ranked pathways of signal transduction involved in platelet activation, although reduced TC and non-HDL levels and diminished macrophage accumulation in lesions are also observed. These effects might also contribute to the diminishment of total lesions area as revealed by Oil Red O staining of this study.

## Conclusion

The current study proposes a learning-based framework called DLDTI for identifying the association of drug targets. The structural characteristics of drug and the characteristics of the protein properties were firstly extracted. An automatic encoder-based model was then proposed for feature selection. Using this feature representation, a convolutional neural network architecture was proposed for predicting the DTI. The advantages of DLDTI were demonstrated by comparing it with three different methods. Experiments on DTI showed that the performance of DLDTI was better than that of the alternative method, which shows that the proposed learning-based framework was properly designed. Consistent with predicted results, the effects and molecular mechanism of TMPZ on atherosclerosis were experimentally confirmed in a novel animal model. With the source code and datasets available at https://github.com/CUMTzackGit/DLDTI, we hope this efficient and feasible computational methods to predict the potential associations between drugs and targets might be of great aid.

## Supplementary information

**Additional file 1:** **Table S1.** Complete results of GO and KEGG analysis.

**Additional file 2:** **Table S2.** Complete results of predicted targets of TMPZ.

## Data Availability

The source code and datasets available at https://github.com/CUMTzackGit/DLDTI.

## Abbreviations

DTI: Drug-target interaction
ROR-γt: Retinoic-acid-receptor-related orphan receptor-gamma t
BLM: Biparticle local model
TMPZ: Tetramethylpyrazine
GIP: Gaussian interaction profile
GF: Graph factorization
SAE: Stack autoencoder
STRING: Search Tool for the Retrieval of Interacting Genes/Proteins
KEGG: Kyoto Encyclopedia of Genes and Genomes
GO: Gene Ontology
Ldlr: Low-density lipoprotein receptor
HCHF: High-cholesterol and high-fat
PCR: Polymerase chain reaction
WT: Wild type
IHC: Immunohistochemistry
SMC: Smooth muscle cell
PRP: Platelet-rich plasma
